# Recent Insights into *Aeromonas salmonicida* and Its Bacteriophages in Aquaculture: A Comprehensive Review

**DOI:** 10.4014/jmb.2005.05040

**Published:** 2020-08-14

**Authors:** Seon Young Park, Jee Eun Han, Hyemin Kwon, Se Chang Park, Ji Hyung Kim

**Affiliations:** 1 Infectious Disease Research Center, Korea Research Institute of Bioscience and Biotechnology, Daejeon 34141, Republic of Korea; 2 Division of Animal and Dairy Sciences, College of Agriculture and Life Science, Chungnam National University, Daejeon 34134, Republic of Korea; 3 Laboratory of Aquatic Biomedicine, College of Veterinary Medicine, Kyungpook National University, Daegu, 41566, Republic of Korea; 4 Laboratory of Aquatic Biomedicine, College of Veterinary Medicine and Research Institute for Veterinary Science, Seoul National University, Seoul 08826, Republic of Korea; 5 Department of Biomolecular Science, KRIBB School of Bioscience, Korea University of Science and Technology (UST), Daejeon 34141, Republic of Korea

**Keywords:** *Aeromonas salmonicida*, antimicrobial resistance, salmonid culture, bacteriophage

## Abstract

The emergence and spread of antimicrobial resistance in pathogenic bacteria of fish and shellfish have caused serious concerns in the aquaculture industry, owing to the potential health risks to humans and animals. Among these bacteria, *Aeromonas salmonicida*, which is one of the most important primary pathogens in salmonids, is responsible for significant economic losses in the global aquaculture industry, especially in salmonid farming because of its severe infectivity and acquisition of antimicrobial resistance. Therefore, interest in the use of alternative approaches to prevent and control *A. salmonicida* infections has increased in recent years, and several applications of bacteriophages (phages) have provided promising results. For several decades, *A. salmonicida* and phages infecting this fish pathogen have been thoroughly investigated in various research areas including aquaculture. The general overview of phage usage to control bacterial diseases in aquaculture, including the general advantages of this strategy, has been clearly described in previous reviews. Therefore, this review specifically focuses on providing insights into the phages infecting *A. salmonicida*, from basic research to biotechnological application in aquaculture, as well as recent advances in the study of *A. salmonicida*.

## Introduction

The genus *Aeromonas* (phylum, Proteobacteria; class, *γ*-Proteobacteria; order, Aeromonadales; and family, *Aeromonadaceae*) comprises a collection of ubiquitous gram-negative bacilli that are widespread in aquatic environments [1]. The taxonomy of this genus is in a continual state of flux as new species are identified by phenotypic and genotypic classifications, and the re-descriptions of the existing taxa are still in progress [2]. In a broad point of view, the genus *Aeromonas* could be divided into motile and non-motile species [3], and a total of 31 species are currently described in the genus [4]. Several motile *Aeromonas* species are known as pathogens of aquatic animals, and interest in this genus has recently increased due to its zoonotic potential [3]. Although the mode of transmission of these pathogens is not clearly understood, some *Aeromonas* species (*e.g.*, *A. hydrophila*, *A. caviae*, *A. dhakensis*, and *A. veronii* biovar. *sobria*) have been recognized as causative agents of human diseases, including gastroenteritis, soft tissue infections, septicemia, peritonitis, pneumonia, and diarrhea [5-9]. Interest in the genus has also increased owing to the emergence of *Aeromonas* isolates that are resistant to commercial antibiotics commonly used in aquaculture and veterinary practice (*e.g.*, *β*-lactams, tetracyclines, and quinolones)[5,10-12].

*Aeromonas salmonicida*, which is known as the only non-motile species in the genus *Aeromonas*, is a primary fish pathogen that causes furunculosis in wild and cultured salmonids as well as bacterial septicemia in a broad variety of fish [13]. From the first findings in the 19th century [14], the species has been considered one of the main bacterial pathogens responsible for significant economic losses in the global aquaculture industry, especially in salmonid culture systems [13]. Previously, this species was thought to be a primary pathogen only in fishes; however, several recent reports have provided evidence on its zoonotic potential [15-17]. Although several antimicrobials and vaccines have been used to prevent or control the onset of disease outbreak in aquaculture [18, 19], furunculosis still occurs as a result of antimicrobial resistance (AMR) or vaccination failure [20, 21]. The emergence and prevalence of AMR in *A. salmonicida* against commercialized antibiotics, such as tetracycline and quinolones, has led to serious concern in the aquaculture industry due to the potential health risks to humans and animals [22-25]. Therefore, interest in alternative approaches that can prevent and control *A. salmonicida* infections has increased in recent years [26].

Bacteriophages (phages) are viruses that solely infect prokaryotic cells, and they are the most abundant living entities on earth [27]. With the global emergence of AMR bacteria, phages have received attention owing to their potential as alternative biocontrol agents, and several phage-based products (*e.g.*, Agriphage, Biotector, BAFADOR, EcoShield, Listex P-100, SalmoFresh, Salmonelex, and PhageGuards) for improved food safety and management of agricultural pathogens have been commercialized [28]. In aquaculture, several studies have verified the promising potential of phages as alternative biocontrol agents against several bacterial pathogens in fish and shellfish [29-31]. In addition, the protective effects of phages against *A. salmonicida* infections have also been established [32-34].

The overview of phage applications to control several bacterial diseases in aquaculture, including their general advantages, has been properly described in previous reviews [29-31,35-38]. This review focuses on the recent advances in the study of *A. salmonicida* to improve the understanding of this important bacterial pathogen in the global salmonid industry. It also provides insights into phages that infect *Aeromonadaceae*, their use in the control of *A. salmonicida*, and the way forward in aquaculture.

## Aeromonas salmonicida

### History of Findings and Classifications

*A. salmonicida* has been recognized as one of the most important fish pathogens for over 100 years. The authentic isolation of *A. salmonicida* and the manifestations of its clinical signs, including furuncle-like swelling and ulcerative lesions on infected fish, were first reported by Emmerich and Weibel [14] during a disease outbreak at a Bavarian brown trout hatchery. Since its first isolation, *A. salmonicida* has been thoroughly investigated and retains its importance as a fish pathogen due to its wide distribution, diverse host range, and devastating economic impact on aquaculture, especially in salmonids [39]. A number of excellent comprehensive reviews on this pathogen, especially on the epizootiological and clinical features including the mechanisms of virulence have been published [39-43].

In the early 20th century, this bacterium was initially referred to as *‘Bacterium’* or *‘Bacillus salmonicida’* [44], but was later designated as *‘Aeromonas salmonicida’* by Griffin *et al*. [45]. The isolates of the bacterium initially appeared to be homogeneous, but an increasing number of studies later reported several isolates with different biological or biochemical properties from those considered ‘typical’ from the 1960s [46]. The isolates of *Aeromonas salmonicida* were classified into two groups, labeled ‘typical’ and ‘atypical’ [47], and were divided into three subspecies, subsp. *salmonicida*, subsp. *achromogenes*, and subsp. *masoucida* [48]. The fourth and fifth subspecies, subsp. *smithia* and subsp. *pectinolytica*, were later proposed by Austin *et al*. [49] and Pavan *et al*. [50], respectively. The List of Prokaryotic Names with Standing in Nomenclature (LPSN; http://www.bacterio.net) [51] now recognizes the five subspecies of *A. salmonicida*: subsp. *salmonicida*, *achromogenes*, *masoucida*, *smithia*, and *pectinolytica*, and currently classifies *A. salmonicida* subsp. *salmonicida* as ‘typical’ and any isolate deviating phenotypically as ‘atypical.’

**Fig. 1:**
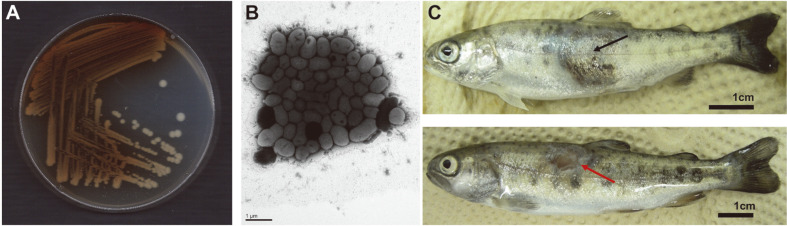
Characteristics of *A. salmonicida* subsp. *salmonicida* (A and B) and its clinical features in salmonid fish (C). (A) Notable brown pigmentation of typical *A. salmonicida* (*A. salmonicida* subsp. *salmonicida* strain AS01 [25]) cultured at 20°C in tryptic soy agar. (**B**) Transmission electron micrograph of *A. salmonicida* subsp. *salmonicida* strain AS01 [25] negatively stained with 2% uranyl acetate (Zeiss TEM EM902 (Zeiss), 80 kV). (**C**) Distinct clinical symptoms of furunculosis caused by *A. salmonicida* subsp. *salmonicida* strain AS01 [25] in experimentally infected rainbow trout (*O. mykiss*). Black and red arrows indicate the notable features of furuncle-like swellings and ulcerative lesions in infected fish, respectively.

Although the typical isolates form a homogeneous group [13,52-54], the phenotypical classification of atypical strains has been relatively ambiguous. This is regardless of the attempts to classify them into several subspecies using single gene (*e.g.*, 16S rRNA, *gyrB*, and *rpoD*) sequencing and molecular fingerprinting tools (*e.g.*, amplified fragment length polymorphism, restriction fragment length polymorphism, and pulsed field gel electrophoresis)[42, 44]. In general, typical strains grow well on blood agar with large colonies, produce a brown diffusible pigment, are *β*-hemolytic, and do not ferment sucrose [55]. Therefore, morphological and biochemical factors, such as pigment production, colony size and growth rate, hemolysis, and sucrose fermentation, are used to distinguish typical and atypical isolates [13, 42, 52, 55] (Fig. 1). However, recent molecular phylogenetic analyses based on multilocus sequence typing [56] and comparative genomic analyses [57, 58] indicated that the five subspecies form a tight phylogenetic cluster, which confirmed their joint classification as subspecies of *A. salmonicida*, and this has facilitated the revision of the complex taxonomy and classification of the species in the genus *Aeromonas*. Moreover, genome-based phylogeny revealed that *A. salmonicida* isolates from different geographical origins are much more diverse than previously thought, and some of these might even be categorized as the sixth new subspecies in the species [57].

### Genome of *A. salmonicida*

Among the genus *Aeromonas*, *A. salmonicida* is one of the most thoroughly genome-sequenced species to date [59]. A total of 68 genome sequences (either complete or draft) are available in the GenBank database (accessed in May 2020), which includes 59 genomes for subsp. *salmonicida*, five for subsp. *achromogenes*, and two for subsp. *masoucida* and *pectinolytica* each (https://www.ncbi.nlm.nih.gov/genome/browse/#!/prokaryotes/540/). With the recent technical advances in genome sequencing, the first complete genome sequence of *A. salmonicida* subsp. *salmonicida* (strain A449) was determined in 2008 [2]. The genomes of *A. salmonicida* subsp. *achromogenes*, subsp. *masoucida*, and subsp. *pectinolytica* were reported in 2013 [60] and 2018 [58, 61], respectively. The A449 genome contained one chromosome (4,702,402 bp encoding 4,388 genes) and two large plasmids (166,749 bp encoding 178 genes and 155,098 bp encoding 164 genes). Notable features were a large inversion in the chromosome and the presence of a Tn*21* composite transposon containing mercury resistance genes and an *In*2 integron coding genes for resistance to streptomycin-spectinomycin, quaternary ammonia compounds, sulphonamides, and chloramphenicol. Moreover, genomic analyses of the A449 strain showed that the chromosome bears two prophages (prophage 1 and 2) sharing structural similarities with the temperate phage φO18P [62], found in *A. media*. The presence of prophages in *A. salmonicida* is of interest in its current genome research because most of the genomes of the isolates from different geographical origins possess the two prophages, while another type of prophage (prophage 3) was recently discovered only in North American isolates [63, 64]. Interestingly, those prophage-containing regions are widely distributed in the various available genomes of other *Aeromonas* spp. in the GenBank database; however, significant similarities between the region and the genomes of other phages are only found in the phages infecting *Enterobacteriaceae* and *Vibrionaceae*, rather than *Aeromonadaceae* [63]. These results suggest that the prophages found in the genome of *A. salmonicida* may have independently evolved from other known phages that infect the bacteria (Ji Hyung Kim, personal communication). Although its exact function has not yet been verified, the prophages in *A. salmonicida* will also be implicated in protection against phages as other prophages in both gram-negative and gram-positive bacteria can provide resistance to infection from other phages by superinfection exclusion systems [65].

### *A. salmonicida* subsp. *salmonicida* and Furunculosis

From its first discovery in 1894, the disease caused by *A. salmonicida* was named ‘furunculosis’ due to its symptom of a furuncle-like swelling, which becomes ulcerative in a later stage of the disease (Fig. 1). However, the discrepancy in the taxonomy of the species has also affected the nomenclature used for the diseases caused by this pathogen. In the pioneer era, the term ‘furunculosis’ was used principally to cover all fish diseases caused by *A. salmonicida* species, even though it was later specifically used for those infections of salmonids which showed the furuncle-like swellings [40]. Ljungberg and Johansson [66] suggested that it was essential from an epizootiological point of view to identify typical and atypical *A. salmonicida* infections as two separate diseases. Subsequently, several diseases caused by atypical isolates in non-salmonid fish have been reported [67], and therefore, only infections caused by *A. salmonicida* subsp. *salmonicida* should be referred to as furunculosis [42, 68].

*A. salmonicida* has extensive host ranges in wild and farmed fish of all ages, and its infections occur in fresh water, brackish, and marine environments [42]. Furthermore, it has been indicated that almost all fish species can serve as reservoirs of infection caused by *A. salmonicida* [69], and salmonids are considered to be the most susceptible to furunculosis, especially Atlantic salmon (*Salmo salar* L.), brook trout (*Salvelinus fontinalis* ), and brown trout (*Salmo trutta* L.). However, rainbow trout (*Oncorhynchus mykiss* ) is considered relatively resistant to this bacterium [40]. Although *A. salmonicida* is regarded mainly as a primary pathogen only in a variety of fishes and not in humans, as they cannot grow at 37°C, several recent reports have indicated that it can cause human infections resulting in septicemia and endocarditis [15-17]. Therefore, in this review, the term furunculosis is used for infections caused by subsp. *salmonicida* and we only focus on the significant features of the subspecies as it concerns the field of aquaculture.

### Emergence of Antimicrobial Resistance (AMR)

The increased frequency of AMR among *A. salmonicida* was first reported in the USA as early as 1967 [70]. Although the emergence and acquisition of resistance in *A. salmonicida* against several classes of antibiotics that are commonly used in aquaculture (*e.g.*, β-lactams, tetracyclines, quinolones, florfenicols, and folate-pathway inhibitors) has been reported, the treatment of the infection is still mostly dependent on the administration of antibiotics [43]. Moreover, several typical strains showing multi-drug resistance have been isolated in recent years [25, 69, 71, 72].

Among the antibiotics utilized in the treatment of furunculosis in aquaculture, the mechanisms of resistance to both tetracycline and quinolone have been thoroughly investigated in the subsp. *salmonicida* [73-75]. Previous studies have indicated that the genetic determinants associated with tetracycline resistance (*e.g.*, *tetA* to *E*) in the species are mostly encoded on the plasmids, and some of the tetracycline-resistant genes are homologous to the ones identified in human and veterinary pathogens [44, 76, 77]. These findings have led to concerns for public health regarding the risk of transfer of AMR to other clinically-relevant pathogens of fish and other animals. In addition, quinolones are the main drug of choice for the treatment of clinical *Aeromonas* infections [78, 79], and they are also used for the treatment of furunculosis and other bacterial fish diseases [80]. In general, quinolone resistance in the genus *Aeromonas* has been associated with 1) plasmid-mediated quinolone resistance (PMQR due to qnr variants) [81], 2) mutations in the quinolone resistance-determining regions (QRDRs) of DNA gyrase and topoisomerase IV [78], and 3) active efflux pump [80]. Among those, the presence of mutations on QRDRs and an active efflux pump belonging to the resistance-nodulation-cell division family that could contribute to quinolone resistance have been reported in quinolone-resistant *A. salmonicida* even recently [25, 78, 80]. Although the significant emergence of PMQR has not been reported in *A. salmonicida*, this species has strong potential to take up and spread the qnr variants to other human and veterinary pathogens as well as other species in the genus *Aeromonas* [81].

### Disease Control and Alternative Approaches

Furunculosis was the first bacterial disease in fish to be treated with antibiotics including sulfonamides and nitrofurans [82], and the outbreaks caused by *A. salmonicida* are usually controlled with chemotherapy [23, 40]. Although other antibiotics effectively control this disease [69], the U.S. Food and Drug Administration imposes stringent restrictions on antibiotics use in the aquaculture industry, and only the use of sulfamerazine, oxytetracycline, and the potentiated sulfonamide Ro5-0037 or ROMET^®^ is approved in the USA [83]. In other countries, several antimicrobial agents have been used to control furunculosis, including chloramphenicol, thiophenicol, furazolidone and oxytetracycline, sulphamerazine, tetracycline, a combination of trimethoprim and sulphonamide, flumequine, oxolinic acid, florfenicol, amoxicillin, and enrofloxacin [40, 69, 84, 85]. Despite the emerging concern on the development of AMR in *A. salmonicida*, the control of pathogens in aquaculture is still mostly reliant on the use of antibiotics.

The global aquaculture industry has adopted vaccination against fish pathogens [86], and several vaccines against typical *A. salmonicida* strains were recently developed to provide long-lasting protection. Their use is promoted in commercial salmonid culture [23, 87, 88]. However, the administration of the vaccines by injection involves substantial regulation in aquaculture (*e.g.* with regard to the numbers, ages, and sizes of fish to be vaccinated) [89] as the vaccines have been linked to a variety of side effects such as impaired growth, inflammation, fibrous adhesions in the internal organ, scarification, and pigment deposition [90-93].

Due to the emergence of AMR and the limitations of vaccination, interest is growing in the use of alternative approaches to prevent and control *A. salmonicida* infections. The use of probiotics has been relatively properly investigated and several bacterial strains, such as *Carnobacterium* [94] and lactic acid bacteria [95, 96], are beneficial for the control of bacterial infections. However, the use of probiotics to control furunculosis is still questionable, as the effects have been variable, and it has been difficult to replicate some results [43, 97]. Some other substances, such as natural products, red clay, and immunostimulants, efficiently inhibit bacterial growth in vitro or protect against *A. salmonicida* infection in fish [21,98-101]. Moreover, phages that infect *A. salmonicida* have received much attention as alternative biocontrol agents against the bacteria. In this review, we present the recent advances in understanding the viral diversity of phages that infect *A. salmonicida* and their applications in the global aquaculture industry.

## Phages Infecting *A. salmonicida* and Their Applications in Aquaculture

### General Description of Phages

Phages are bacterial viruses that infect bacterial cells, disrupt bacterial metabolism, and cause the bacterium to lyse. They are the most abundant living entities on earth, and they play major roles in bacterial ecology, adaptation, evolution, and pathogenesis [102]. Phages are common in soils (approximately 10^7^ to 10^9^ virions/g) and highly abundant in fresh water as well as marine ecosystems (approximately 10^7^ virions/ml), and their total number on earth was once estimated at 10^31^ virions [103].

The discovery of phages was initially reported by Ernest H. Hankin in 1896 when the first evidence for a viral-like agent with antibacterial properties against *Vibrio cholera* was observed [27]. Afterwards, phages were rediscovered twice at the beginning of the 20th century. Frederick W. Twort, an English medical bacteriologist, described a marked antibacterial activity in *Micrococcus* by an unknown agent in 1915, and 2 years later, phages were “officially” discovered by Felix H. d’Herelle, a French-Canadian microbiologist at the Institut Pasteur [104]. He discovered the destruction of *Shigella* in broth culture, recognized the viral nature of this phenomenon and suggested the term ‘bacteriophage’ [105]. The viral nature of phages was recognized in 1940 with the development of the electron microscope, and the basis of the present phage classification was proposed by Bradley in 1967 [106] as six types—tailed phages, filamentous phages, and icosahedral phages with single-stranded (ss) DNA or ssRNA.

At present, the classification and naming of phages is maintained by the Bacterial and Archaeal Subcommittee within the International Committee on Taxonomy of Viruses (ICTV) [107]. Phages have been classified based on the various viral properties such as virion morphology (the structure of the viral capsid and presence of envelops), genome type of the virus (ssDNA, dsDNA, ssRNA, or dsRNA), the species of host bacteria, life cycle (lytic or lysogenic), and genome similarity [108]. However, due to the complexity of features that contribute to the taxonomy of phages, their classification is complex and still evolving. In 1971, ICTV classified phages into only six genera (T4, λ, φX174, MS2, fd, and PM2) [109]. Later, the classification of phages was revised based on the capsid morphology of the virion, and the classes include polyhedral (*Microviridae, Corticoviridae, Tectiviridae, Leviviridae* and *Cystoviridae*), filamentous (*Inoviridae*), pleomorphic (*Plasmaviridae*), and tailed (*Caudovirales*)[110]. Since then, new phage groups have been continuously added, particularly tailed phages containing dsDNA genome. In addition, only a few phage types containing lipid or with a lipid-containing envelop have been found [111]. The 1999 ICTV report classified tailed phages into three families, 16 genera, and 30 species, but its 2018 report revised them into five families, 26 subfamilies, 363 genera, and 1,320 species (https://talk.ictvonline.org/taxonomy/p/taxonomy_releases). Recent advances in next-generation sequencing (NGS) technologies unveiled the ‘hidden’ genomic and metagenomic sequence of unknown phages, but unfortunately, a systematic classification of these phage genomes into the ICTV scheme is not available due to lack of related biological properties [112-114]. Therefore, taxonomical revision based on the genomic information of phages has become indispensable, and modernized comprehensive guidelines for phage classification have been recently suggested, which is expected to cause a substantial increase in the list of virus taxa in the coming years [115, 116].

As for their bactericidal mechanisms, phages are known to have two possible life cycles; the ‘lytic’ (or virulent) and ‘lysogenic’ (or temperate) cycles [117]. Lytic phages rapidly multiply and kill the host cell at the end of the replication cycle. Moreover, temperate phages that undergo the lysogenic cycle persist in a lysogenic state, whereby the phage genome can exist indefinitely when inserted in bacterial chromosome (known as the prophage state). For example, the lysogenic life cycle of λ phage ensures the replication of the integrated prophage along with the bacterial genome for many generations. When induction occurs through DNA damage (UV irradiation or exposure to mutagens), which signifies the imminent death of the host, the phage switches to the lytic cycle which results in the release of new phage particles. Interestingly, it has been reported that temperate phages transfer foreign genes into their host bacteria, including toxins and other virulence determinants [118]. In fact, some prophages can change non-pathogenic bacteria to pathogenic ones through lysogenic conversion mechanism, which is now considered the most ostensible contribution to their pathogenesis. In fact, many of the toxins that are responsible for diseases such as diphtheria, cholera, hemolytic-uremic syndrome, botulism, or food poisoning are encoded by temperate phages, and in some cases, their expression also relies on the phages by linking of their regulation to the lytic cycle [119]. Several examples of toxin gene and pathogenic island insertions of temperate phage to host bacterium have been comprehensively reviewed in the literature [119, 120]. Moreover, the role of phage-mediated transduction and lysogenic conversion in the spread of AMR determinants is a recent topic of research although these genes are much more often found in conjugative genetic elements than in phages. Moreover, several cases on the presence of AMR genes in the phages and prophages have been identified, thus suggesting that phages could play an important role in their transmission between bacterial communities and deserve further attention in the future [121, 122].

In addition, bacteria and their associated phages undergo continuous cycles of evolution to generate resistance to each other through an antagonistic, microscopic arms race [65, 123]. The innate and adaptive bacterial resistance (or immune) systems discovered in response to invading phages are enormously diverse, and much still remains to be discovered [124]. Currently described bacterial innate phage-resistnace mechanisms involve i) aversion of phage adsorption or ii) blockage of phage DNA entry. When these mechanisms fail bacterial protection, iii) the abortive infection triggers the suicide of phage-infected bacterial cells by preventing replication of progeny virus, which finally benefits the bacterial population adjacent to the infected ones [124]. On the other hand, iv) a Clustered, Regularly Interspaced, Short Palindromic Repeat (CRISPR) locus is the only known adaptive immune system in bacteria; a short phage-originated DNA fragment is integrated into the CRISPR loci and finally produces specific immunity against the invading phage [65]. In response to these bacterial phage-immune systems, phages simultaneously evolved their own strategies (such as the anti-CRISPR systems) to avoid, circumvent, or subvert those antiviral mechanisms to successfully complete their lytic cycles [124].

Naturally, phages are found wherever their host bacteria exist [125], and there are several reviews that have focused on the viral communities from soil, water, and host-associated systems [126-128]. The prevalence of phage-mediated lysogenic (rather than lytic) infections in the aquatic environment is still controversial, although more than 90% of known phages are considered temperate in nature [129]. Other more recent studies have reported lower levels of lysogeny in aquatic microbial populations, ranging from 2% [130] to 47% [131]. Moreover, recent NGS approaches in microbial genomics have revealed that temperate phages are prevalent in bacteria in every ecosystem and organism, and about half of those genomes contain temperate phages [132, 133], thus indicating that a large percentage of existing phages are lysogenic. However, temperate phages are not suitable candidates for phage therapy because they may not immediately kill the host bacteria and transfer foreign genes into the host as previously described [119, 120]. Therefore, we will mainly focus on the lytic phages that infect aquatic pathogens including *A. salmonicida* and their potential applications in aquaculture systems in the subsequent sections of this review.

### Therapeutic Application of Phages

Even though phages were discovered in the early 20th century, research on their possible therapeutic applications against infectious bacterial diseases in the past half century has been limited [134]. This poor understanding of bacterial pathogenesis and phage-host interactions has led to a succession of badly designed and executed experiments. Furthermore, with the advent of antibiotic therapy, the use of phages became underexplored, especially after World War II. The discovery of antibiotics diverted research attention from phage therapy, mainly in the USA and Western Europe in the 1940s. However, the use of phage therapy has persisted without interruption in Eastern Europe and the Soviet Union, and a number of companies have even commercialized phages [135]. In the past, with regards to human health, phage was commercialized and administered in Eastern Europe and the Soviet Union orally, topically, or systemically to treat a wide variety of human infections (suppurative wound, gastroenteritis, sepsis, osteomyelitis, dermatitis, emphysema, and pneumonia) in both adults and children with promising results. In thehe previous enthusiasm on the application of phages to prevent and treat bacterial infections in human was reinvigorated [136, 137]. In 1992, the studies of Soothill, using mice and farm animals infected with *E. coli*, showed that phages could be used for both the treatment and prevention of bacterial infections [138]. Since then, several other Polish and Soviet Union study groups have presented successful clinical applications of phages against drug-resistant bacterial infections in humans and animal models [136]. The therapeutic efficacy of phages against infectious diseases caused by *Pseudomonas aeruginosa, Staphylococcus aureus* (including MRSA), *E. coli*, *Enterococcus faecium* (including VRE), *Streptococcus pneumoniae, Helicobacter pylori, Klebsiella pneumoniae*, and *Salmonella enteritidis* have been demonstrated in various experimental animal models [134,137,139-146]. Moreover, in recent decades, the emergence of antibiotic-resistant bacteria has substantially enhanced the interest of researchers in phage therapy, even in the USA and Western Europe. Nowadays, more than a dozen of companies and universities are working on phage therapy for humans, using current standards of clinical and microbiological research [28, 147, 148].

Numerous recent studies have evaluated phages as biocontrol agents in food [146,149-151] and plants [152], and for wastewater treatment [153]. Bacterial diseases are a major problem in aquaculture [134, 154, 155]. The increasing problems related to worldwide emergence of AMR in common pathogenic bacteria and the concerns about its spread in aquaculture environments demand alternative control methods for bacterial pathogens in fish and shellfish. In aquaculture, phage therapy is a potentially viable alternative to antibiotics in the control of indigenous and non-indigenous bacterial disease in farmed fish and shellfish [29-31]. In addition, some studies on phages have involved the identification of phages for use in bacterial typing schemes or for characterization, including investigation of their potential role in virulence [155-157]. Remarkably, several studies have demonstrated the ability of phages to prevent or control bacterial infections associated with *Vibrio* spp., *Flavobacterium* spp., *Aeromonas* spp., *Pseudomonas* spp., and *Lactococcus* spp. in aquaculture in fish or shellfish [29-31]. In the same manner, there have been several reports of the antibacterial effects of phages against *A. salmonicida* infections in aquaculture [32-34].

### Phages Infecting *Aeromonadaceae* (Especially *A. salmonicida*)

Historically, phages that infect *Aeromonadaceae* (hereinafter referred to as *Aeromonas* phages) have been studied for a relatively long time compared to other bacterial species, and a large number of phages have been isolated and characterized. The first *Aeromonas* phage with morphological features based on the electron microscope was reported in 1965 [158]. Its host, which was identified as an *Acetobacter* sp., was later reclassified as *Aeromonas* sp. [159]. Subsequently, Paterson isolated nine *Aeromonas* phages infecting *A. salmonicida* from trout ponds and fish hatcheries and described the characteristics of some selected phage isolates [160]. A halophilic and psychrophilic phage, specific for a marine *Aeromonas* spp., was isolated from seawater collected at a depth of 825 m [161]. In 1971, 35 *Aeromonas* phages infecting *A. salmonicida*, isolated from sewage, surface water, fish hatcheries, and lysogenic bacteria, were characterized by serology and various biological criteria. Sixteen of these phages were studied by electron microscopy and were divided into three morphological groups [162]. During the last decades, a number of lytic and/or lysogenic phages infecting *A. salmonicida* have been described and characterized [159,163-167]. However, their classification depended largely on morphology and serological data due to the absence of the physicochemical and genetic properties of the isolated phages [159]. In fact, many *Aeromonas* phages were described without accurate morphological micrographs, until the first morphological characteristics of about 35 *Aeromonas* phages, mostly those that infect *A. salmonicida*, were thoroughly investigated by Ackermann [159]. In the more recent review by Ackermann [102], a total of 43 previously isolated *Aeromonas* phages (mainly infecting *A. hydrophila* and *A. salmonicida*) were reinvestigated, and all them were morphologically classified as tailed members of *Caudovirales* (*Myoviridae* (*n* = 33), *Siphoviridae* (*n* = 7), and (*Podoviridae* (*n* = 3) (recently classified as (*Autographiviridae*) [114]). Furthermore, most of the phages that belong to the family *Myoviridae* were classified into P1-, P2-, and T4-like viruses in the VIII^th^ ICTV Report (http://www.ictvdb.org/Ictv/index.htm) [168].

The recent advances in genome sequencing technology and its adaptations in the phage taxonomy facilitates the investigation of the morphology and genetic functions of *Myoviridae* phages infecting *E. coli* and other gram-negative bacteria (especially T4 and T4-like viruses), and this provides an attractive model for the study of comparative genomics and evolution of phages [169, 170]. According to the recent viral taxonomy of the IX^th^ ICTV Report released in 2019 (https://talk.ictvonline.org/ictv-reports/ictv_9th_report/dsdna-viruses-2011/w/dsdna_viruses/68/myoviridae), the family *Myoviridae* has been divided into five subfamilies. Moreover, detailed classification of previously isolated phages using a range of complementary sequence analysis tools as well as phylogenetic methods is still in progress [171]. In this respect, recent studies on *Aeromonas* phages have also focused on *Myoviridae* phages and have included extensive genomic investigations to elucidate their plasticity [34,169,170,172-185]. A total of 29 complete genomes of phages that infect *A. salmonicida* are currently available in the GenBank database (accessed in May, 2020) (Table 1), and most of the sequenced *Aeromonas* phages were classified as *Myoviridae* (*n* = 26) with the exception of two species in the *Autographiviridae* family and one species in the *Siphoviridae* family. Based on this information, it can be assumed that *Aeromonas* phages belonging to the *Myoviridae* family are more prevalent than the other phages. In addition, further research on *Aeromonas* phages to unveil their diversity in aquatic environments and explore their biotechnological applications in aquaculture is expected. The recent advances in metagenomics have enabled us to understand the diversity of the viral community and obtain various in silico genomes of phages from aquatic environments [186, 187]. Thus, the discovery of several novel *Aeromonas* phage genomes is expected in the future. Although much effort has been made to understand phage adsorption and identify receptors involved in the lytic cycle of phages in various bacterial species [188], studies on finding bacterial receptors and understanding the resistance mechanism in *A. salmonicida* against its phages are still elusive and much still remains to be discovered [189]. Among the large numbers of isolated phages infecting *A. salmonicida*, only three studies have characterized the bacterial receptor of the specific phages; lipid A of the lipopolysaccharide was identified as a receptor for the phage 55R-1, and A-layer (or S-layer), was also reported as a receptor for phages TP446 and SW69-9, respectively [189]. Based on these results, it can be suggested that modifications to the receptors in the outer membrane protein might be among the most important mechanisms of resistance to phages for *A. salmonicida*. Furthermore, the phage receptors in this species could be more diverse than previously thought. However, much work still needs to be done in future.

### Application of Phages Infecting *A. salmonicida* and Future Perspectives

A number of phages infecting various bacterial pathogens of fish and shellfish have been isolated and the therapeutic (or prophylactic) application of those phages in aquatic animal models has demonstrated their promising potential as alternative antimicrobial agents in aquaculture [29, 30]. Although *Aeromonas* spp. are recognized as the third most targeted aquatic bacterial pathogens in phage application research [31], historically, the genus was the first reported target of phage application in aquaculture [190]. Since then, numerous studies, mainly focused on *A. hydrophila* and *A. salmonicida*, have evaluated the therapeutic (or prophylactic) potential of *Aeromonas* phages in various fish species, and the phages have produced promising results as alternative biocontrol agents in aquaculture.

*A. salmonicida* is principally recognized in cultured salmonid species as a major pathogenic bacteria causing significant economic losses in the global salmonid farming industry [191]. Therefore, numerous studies have been conducted to evaluate antimicrobial activity using various *A. salmonicida* strains and their phages (including phage cocktails) and have shown sufficient biocontrol efficacy of phages at the in vitro and/or in vivo levels [32-34,180,182,192-194] (Table 2). Among them, therapeutic (or prophylactic) applications of phages against *A. salmonicida* have been conducted to control furnunculosis in farmed brook trout (*S. fontinalis*) [32], Atlantic salmon (*S. salar*) [192], and rainbow trout (*O. mykiss*) [33]. Although Verner-Jeffreys *et al*. did not find any protective effects against *A. salmonicida* in phage-treated fish [192], the other two studies showed clear differences between the phage-treated group and the control group. Imbeault *et al*. reported that the administration of phage HER110 delayed onset of furunculosis by 7 d with reduced mortality rates of the fish from 100% to 10% [32]. Moreover, administration of phage PAS-1 in rainbow trout model showed notable protective effects against *A. salmonicida* infection with increased survival rates (0% to 30%) [33]. In addition, the protective effect of phage against *A. salmonicida* was also verified in Senegalese sole (*Solea senegalensis*), showing significantly reduced mortality (36% to 0%) [34]. Due to the differences in *Aeromonas* phages and fish species, it is difficult to generalize the findings of different studies on the effects of phages on bacterial infections in fish. However, previous studies have generally demonstrated the protective effects of phages against *A. salmonicida* infections and their potential efficacy to control furunculosis in aquaculture.

Salmonid farming is currently a major global industry and its growth has been largely supported by the intensification of fish culture. However, the increased level of mortality associated with *A. salmonicida* and the prevalence of AMR have placed ever-growing importance on the development of alternative control methods against the bacteria [195]. Among the suggested alternatives, the use of phages to control *A. salmonicida* has shown the most beneficial characteristics, but there are still several limitations to be addressed. The general advantages and limitations of phage applications in aquaculture have been extensively described in previous reviews [29-31,35-38]. Nevertheless, based on our experience, we highlighted below the challenges associated with the industrial use of *Aeromonas* phages for the control of *A. salmonicida* in salmonid farming and how such challenges can be overcome.

1. In terms of phages:
A. Finding *Aeromonas* phages with broad infectivity is the first step. In our experience, some isolated *Aeromonas* phages were able to infect several different species of *Aeromonas* strains as well as other subspecies of *A. salmonicida* [178, 180].
B. In aquaculture, the importance of phage genome sequencing tends to be overlooked. The safety of isolated phages should be examined at the genome level, and phages with genes related to lysogenic conversion (such as *integrase*) or potentially damaging genetic determinants (toxins or AMR genes) should be excluded from further application [196].
C. The emergence of phage-resistant bacteria is one of the major limitations of phage application, and alternatively, a combination of different phages (phage cocktail) or a combination of a phage with antibiotics, preservative, or disinfectants is recommended [35].
2. In terms of *A. salmonicida*:
A. Regular surveillance studies of *A. salmonicida* isolates from cultured fish will be necessary. For field application, the infectivity of *Aeromonas* phages against bacteria isolated close to the onset of disease should be verified.
B. In aquaculture, the importance of understanding the interactions between host microbe and phage also tends to be overlooked. Understanding the bacterial phage-resistance mechanisms and identifying receptors on the selected phage will be crucial for its successful application in aquaculture [189].
3. In terms of salmonid fish:
A. Although an anti-phage immune response limiting the efficacy of phage therapy has been identified in humans [197], only limited studies on the impact of this immunomodulation during phage administration have been conducted in fish [33, 198]. More studies are required to evaluate this anti-phage response in salmonid fish.
B. For the application of phages in aquaculture, selection of methods as well as timing and dosage (multiplicity of infection) of phage administration are considered very important factors [31]. However, prophylactic use of phages, followed by eventual therapeutic use, seems to be the best application strategy for *Aeromonas* phages in salmonid culture.

Numerous recent applications of phages have shown promising protective efficacy with several advantages over antibiotics. However, more studies geared towards the optimization of phage application under field (or farm scale) conditions rather than lab-scale conditions are required [199]. Moreover, understanding the natural mechanisms that contribute to the emergence of phage-resistant strains and identifying the potential bacterial receptors of specific phages will be crucial to provide a successful path to phage biocontrol as an alternative treatment method in aquaculture [189]. Similar to antimicrobials, the initial idea of phage therapy was for the treatment of diseases; however, considering the nature of the aquaculture industry, future phage research that guarantees industrialization should rather focus on its prophylactic use to reduce potential pathogen loads that can cause severe outbreaks. In addition, there is still a need to overcome the understandable stigma among producers and consumers regarding the safety of phages despite the certification by the regulatory bodies [200]. Notwithstanding the recent increase in scientific interest in the industrial application of phage, only a small number of private companies have publicized their intention to work on phage-based solutions for aquaculture and few products have been commercially released [31]. Therefore, additional efforts are required to assess the understanding of producer and consumer followed by educational campaigns to raise the awareness and acceptance on the use of phages in aquaculture. Although several limitations are still associated with the use of phages, they still have undeniable advantages over the other alternatives. Therefore, exploring phage-based products is now more necessary than ever as the aquaculture industry is presently facing increasing problems with AMR pathogens including *A. salmonicida*.

### Concluding Remarks

Salmonid farming today is a major global industry; however, it is increasingly threatened by economic losses associated with *A. salmonicida*. From the first findings in the 19^th^ century, *A. salmonicida* has been considered a major bacterial pathogen in aquaculture, especially in salmonid culture. Due to its taxonomical complexity and severe pathogenicity in cultured fish, researchers have been extensively investigating *A. salmonicida* for several decades. The emergence and spread of AMR in *A. salmonicida* is of great concern in aquaculture, and interest in the use of alternative approaches to prevent and control infection has increased in recent years.

Several studies have verified the promising potential of phages as biocontrol agents against various bacterial pathogens including fish and shellfish, and the genus Aeromonas, which was the first reported target for the application of phages in aquaculture, has become the third most targeted pathogen in phage application research. Historically, *Aeromonas* phages have been studied for more than 50 years, and a large number of phages have been isolated and investigated, mainly on their morphological and biological diversity. Moreover, recent studies on *Aeromonas* phages have focused on extensive genomic investigations to elucidate their plasticity, and further studies are expected to unveil the diversity of *Aeromonas* phages in aquatic environments. In aquaculture, the biocontrol potential of *Aeromonas* phages against *A. salmonicida* has been verified in cultured salmonid species and shows highly promising characteristics; however, more studies are required to optimize phage application under field (or farm-scale) conditions and to understand the interactions between host fish, bacteria and phage. The initial idea of phage therapy was for the treatment of diseases; however, considering the nature of the aquaculture industry, future phage research should focus on prophylactic application to reduce the potential load of pathogens, including *A. salmonicida*, to prevent severe outbreaks.

## Figures and Tables

**Table 1:** List of genomes of phages infecting *A. salmonicida* available in the GenBank database.

Name of phage	Family	Host	Isolation source /country	Genome size (bp)	GenBank No.	Reference
51	*Myoviridae*	*A. salmonicida*	Water/France	43,551	KY290953.1	[65]
	*(Popoffvirus)*					
56	*Myoviridae*	*A. salmonicida*	Water/France	43,551	KY290954.1	[65]
	*(Popoffvirus)*					
59.1	*Myoviridae*	*A. salmonicida*	Water/Canada	46,057	KY290950.1	[65]
3	*Myoviridae*	*A. salmonicida*	Water/France	46,349	KY290947.1	[65]
Asp37	*Myoviridae*	*A. salmonicida*	Water/Canada	47,977	KY290949.1	[65]
32	*Myoviridae*	*A. salmonicida*	Water/France	48,252	KY290952.1	[65]
31.2	*Myoviridae*	*A. salmonicida*	Water/France	172,957	KY290951.1	[65]
	*(Biquartavirus)*					
SW69-9	*Myoviridae*	*A. salmonicida*	Water/Canada	173,097	KY290958.1	[65]
	*(Biquartavirus)*					
L9-6	*Myoviridae*	*A. salmonicida*	Water/Canada	173,578	KY290956.1	[65]
	*(Biquartavirus)*					
44RR2.8t.2	*Myoviridae*	*A. salmonicida*	Water/Canada	173,590	KY290948.1	[65]
	*(Biquartavirus)*					
65.2	*Myoviridae*	*A. salmonicida*	Water/France	236,567	KY290955.1	[65]
	*(Tevenvirinae)*					
Aes508	*Myoviridae*	*A. salmonicida*	N/A/USA	160,646	NC_019543.1	[65]
	*(Tulanevirus)*					
AS-szw	*Myoviridae*	*A. salmonicida*	Water/China	29,957	MF498773.1	[183]
	*(Tevenvirinae)*	subsp. *salmonicida*				
AS-gz	*Myoviridae*	*A. salmonicida*	Water/China	162,422	NC_042019.1	[183]
	*(Tulanevirus)*	subsp. *salmonicida*				
AS-zj	*Myoviridae*	*A. salmonicida*	Water/China	229,929	MF448340.1	[183]
	*(Tulanevirus)*	subsp. *salmonicida*				
AS-sw	*Myoviridae*	*A. salmonicida*	Water/China	230,024	MF498775.1	[183]
	*(Tulanevirus)*	subsp. *salmonicida*				
AS-yj	*Myoviridae*	*A. salmonicida*	Water/China	230,183	MF498774.1	[183]
	*(Tulanevirus)*	subsp. *salmonicida*				
25	*Myoviridae*	*A. salmonicida*	Fish farm/France	161,475	NC_008208.1	[170]
	*(Tulanevirus)*					
31	*Myoviridae*	*A. salmonicida*	Fish farm/France	172,963	NC_007022.1	[170]
	*(Biquartavirus)*					
65	*Myoviridae*	*A. salmonicida*	River/France	235,229	NC_015251.1	[170]
	*(Tevenvirinae)*					
PX29	*Myoviridae*	*A. salmonicida*	Sewage/USA	222,006	NC_023688.1	[202]
	*(Tevenvirinae)*					
vB_AsaM-56	*Myoviridae*	*A. salmonicida*	Freshwater, France	433,551	NC_019527.1	[177]
	*(Popoffvirus)*					
44RR2.8t	*Myoviridae*	*A. salmonicida*	Fish farm/Canada	173,591	NC_005135.1	[173]
	*(Biquartavirus)*					
AsXd-1	*Siphoviridae*	*A. salmonicida*	Wastewater/China	39,014	MH178096.1	[184]
phiAS4	*Myoviridae*	*A. salmonicida*	River/Korea	163,875	HM452125.1	[178]
	*(Tulanevirus)*					
phiAS5	*Myoviridae*	*A. salmonicida*	River/Korea	225,268	NC_014636.1	[179]
	*(Tevenvirinae)*					
phiAS7	*Autographiviridae*	*A. salmonicida*	Fish farm/Korea	41,572	NC_019528.1	[180]
		subsp. *salmonicida*				
Asfd_1	*Myoviridae*	*A. salmonicida*	Sewage/China	168,962	MK577502.1	[186]
	*(Biquartavirus)*					
PS	*Autographiviridae*	*A. salmonicida*	Sewage/India	41,082	MT259468.1	N/A
AS-A	*Myoviridae*	*A. salmonicida*	Sewage/Portugal	N/A	N/A	[34, 185]
PAS-1	*Myoviridae*	*A. salmonicida*	Fish farm/Korea	N/A	JF342683.1-	[181]
		subsp. *salmonicida*			JF342690.1	

^*^N/A, not available.

^*^The genome data of phages in the GenBank database were accessed in May 2020.

**Table 2:** List of phage-biocontrol approaches against *A. salmonicida* in aquaculture.

Bacterial strain	Fish species	Challenge	Phages	Administration (efficient MOI^*^)	Biocontrol potential	Results	Reference
HER1107	Brook trout (*S. fontinalis*)	Immersion	HER 110	Immersion (1)	Yes	Delayed onset of furunculosis by 7 d and reduced total mortality rates from 100% to 10%	[32]
Cefas 78027	Atlantic salmon (*S. salar*)	Cohabitation or I.P. injection	O, R, and B	Various methods (Various MOIs^**^)	No	No protective effects were observed	[193]
AS01, ATCC 27013	ND (in vitro only)	ND (in vitro only)	PAS-1	ND (0.1)	Yes	Bacterial growths were apparently retarded until 24 h	[181]
AS05	Rainbow trout (*O. mykiss*)	I.M. injection	PAS-1	I.M. injection (10,000)	Yes	Notable protective effects with increased survival rates (0% to 30%). Neutralizing activity against PAS-1 was detected in the phage-treated fish	[33]
CECT 894	Senegalese sole (S. senegalensis)	Immersion	AS-A	Immersion (100)	Yes	Inhibition of bacterial growth both in the seawater and batch cultures with fish, and no mortality was observed compared to control group (36%).	[34]
AS-sz	ND (in vitro only)	ND (in vitro only)	AS-szw, AS-yj, AS-zj, AS-sw, and AS-gz	ND (0.01)	Yes	Efficient phage cocktail was designed and its synergetic antimicrobial activity was confirmed	[183]
CECT 894	ND (in vitro only)	ND (in vitro only)	AS-A, AS-D, and AS-E	ND (100)	Yes	Efficient phage cocktail was designed and its synergetic antimicrobial activity was confirmed	[194]
Unnamed isolate	ND (in vitro only)	ND (in vitro only)	ASP-1	ND (0.01)	Yes	Bacterial growths were apparently retarded until 12 h	[195]

^*^MOI, Multiplicity of infection.

^**^The exact administration methods of phages and MOIs can be found in Verner-Jeffreys *et al*. [193].
